# Immune recurrence score using 7 immunoregulatory protein expressions can predict recurrence in stage I–III breast cancer patients

**DOI:** 10.1038/s41416-019-0511-9

**Published:** 2019-07-11

**Authors:** Dae-Won Lee, Han Suk Ryu, Min-Sun Jin, Kyung-Hun Lee, Koung Jin Suh, Jeonghwan Youk, Jung Youn Kim, Ahrum Min, Han-Byoel Lee, Hyeong-Gon Moon, Tae-Yong Kim, Sae-Won Han, Do-Youn Oh, Wonshik Han, In Ae Park, Dong-Young Noh, Seock-Ah Im

**Affiliations:** 1 Department of Internal Medicine, Seoul National University Hospital, Seoul, Korea; 2 Department of Pathology, Seoul National University Hospital, Seoul, Korea; 3 Department of Pathology, Bucheon St. Mary’s Hospital, Bucheon, Korea; 4 Cancer Research Institute, Seoul National University College of Medicine, Seoul, Korea; 5 Biomedical Research Institute, Seoul National University Hospital, Seoul, Korea; 6 Department of Internal Medicine, Seoul National University Bundang Hospital, Seongnam, Korea; 7 Department of Surgery, Seoul National University Hospital, Seoul, Korea

**Keywords:** Breast cancer, Immunology

## Abstract

**Background:**

Immune cells in the tumour microenvironment play an essential role in tumorigenesis. This study aimed to evaluate the immunoregulatory protein expression of breast cancer and reveal their prognostic role.

**Methods:**

Expression of 10 immune markers (PD-1/PD-L1/PD-L2/IDO/TIM-3/OX40/OX40L/B7-H2/ B7-H3/B7-H4) with known/possible clinical relevance was identified in stromal tumour-infiltrating lymphocytes or tumour tissue of stage I–III breast cancer patients.

**Results:**

A total of 392 patients, including 271(69.1%) luminal A, 36(9.2%) luminal B, 32(8.2%) HER2-positive and 53(13.5%) triple negative disease, were included. Expression of PD-1 and PD-L1 was higher in HER2-positive and triple negative disease. By contrast, expression of TIM-3, OX40 and OX40L were higher in luminal disease. We devised an immune recurrence score (IRS) using seven markers with prognostic value (B7-H2/B7-H3/B7-H4/OX40/OX40L/PD-L1/PD-L2). Patients were classified as high-risk (7.9%), intermediate-risk (67.6%), or low-risk (24.5%). In the multivariate analysis, IRS low-risk (adjusted HR 0.14, *p* = 0.001) and intermediate-risk (adjusted HR 0.32, *p* = 0.002) had significantly lower risk of recurrence compared with high-risk. The prognostic role of IRS was maintained in both luminal A and non-luminal A patients.

**Conclusions:**

This study identified immunoregulatory protein expression of breast cancer patients using 10 immune markers. In addition, we devised an IRS which may predict recurrence in stage I-III breast cancer patients.

## Background

Besides the classical six hallmarks of cancer proposed by Hanahan et al., avoiding immune destruction has emerged as a new hallmark of cancer.^1^ Complex interactions between cancer and immune system exist, which are controlled by multiple mechanisms. Among these mechanisms, cancer cells can avoid immune destruction by alternating the immune checkpoint pathway. Recently, immune checkpoint blockades targeting cytotoxic T-lymphocyte-associated antigen (CTLA)-4, programmed death 1 (PD-1), and programmed death ligand 1 (PD-L1) have shown dramatic effect in various tumour types, including melanoma, non-small cell lung cancer and urothelial carcinoma.^2–5^

Trials evaluating the efficacy of immunotherapy agents in breast cancer have been actively investigated and are ongoing. Efficacy of anti PD-1 antibody, pembrolizumab, was investigated in the KEYNOTE-012 study. The overall response rate (ORR) of pembrolizumab was 18.5% of the 27 advanced triple negative breast cancer patients with at least 1% PD-L1 expression.^6^ In a phase 1b JAVELIN solid tumour trial, anti-PD-L1 antibody, avelumab, showed ORR of 4.8% among 168 breast cancer patients.^7^ Although the ORR was low in unselected patients, ORR was 33.3% in 12 patients who had PD-L1 expressing immune cells within the tumour. Recently, the result of IMpassion 130 study showed that atezolizumab plus nab-paclitaxel is superior to nab-paclitaxel monotherapy in metastatic triple-negative breast cancer.^8^ These evidences show that immunotherapy agents may be effective in breast cancer patients. However, only a proportion of breast cancer patients received benefit, and more knowledge on the interaction between immune cells and breast cancer cells needs to be investigated.

Many types of immune cells, such as myeloid lineage leucocytes, macrophages, helper T cells, cytotoxic T cells, regulatory T cells, B cells and dendritic cells, infiltrate tumour microenvironment.^9^ Although each subsets of immune cells have different effect on tumour suppression or progression, tumour-infiltrating lymphocytes (TILs) as a whole showed a positive prognostic and predictive role in breast cancer patients.^9–11^ While immunologic characteristics may affect the response of breast cancer to immunotherapy agents, the immunologic characteristic of breast cancer has not been clearly identified. The purpose of this study was to reveal the immunoregulatory protein expression of breast cancer and to investigate their prognostic role. We selected 10 immune markers and performed immunohistochemical staining in 392 stage I–III breast cancer patients who had undergone curative surgery.

## Methods

### Study population

This study included 392 pathologically proven breast cancer patients who received curative resection at Seoul National University Hospital (SNUH, Seoul, Korea) between January 2008 and December 2008. Primary treatments included radical mastectomy, modified radical mastectomy and breast-conserving surgery with concomitant sentinel lymph node biopsy or axillary lymph node dissection. Adjuvant chemotherapy and radiotherapy were administered at the discretion of treating physician. Patients were excluded if they received neoadjuvant chemotherapy. Eligible patients were identified from the electronic database and medical charts were reviewed using the electronic medical record system of SNUH. The study protocol was reviewed and approved by an institutional review board of SNUH [H-1409-017-607]. This study was carried out in accordance with the recommendations of the Declaration of Helsinki for biomedical research involving human subjects.

### Immunohistochemical analysis of immune markers

Immunohistochemical staining was performed with formalin-fixed, paraffin-embedded tissue using Benchmark automatic immunostaining device (Ventana, Arizona, USA) as previously described.^12^ The primary antibodies were diluted as follows: oestrogen receptor (ER) (1D5; Novocastra Laboratories, Newcastle, UK), 1:100; progesterone receptors (PR) (PgR636; DAKO, Hamburg, Germany), 1:200; human epidermal growth factor receptor 2 (HER2) (Ventana, Arizona, USA), 1:1; PD-1 (Cell Marque, California, USA), 1:20; PD-L1 (B7-H1) (Cell Signaling, Massachusetts, USA), 1:100; PD-L2 (B7-DC) (Sigma-Aldrich, Missouri, USA), 1:500; IDO (Millipore-Sigma, Massachusetts, USA), 1:30; TIM-3 (Abbexa Ltd, Cambridge, UK), 1:550; OX40 (Novus Biologicals, Colorado, USA), 1:125; OX40L (Millipore-Sigma, Massachusetts, USA), 1:30; B7-H2 (Novus Biologicals, Colorado, USA), 1:300; B7-H3 (Cell Signaling, Massachusetts, USA), 1:50; B7-H4 (Cell Signaling, Massachusetts, USA), 1:50. Nuclear expression of tumour cells was interpreted as positive for ER and PR, while membrane staining of tumour cells was considered positive for HER2. Expression of PD-1, PD-L1 and PD-L2 was counted in tumour cells and stromal TILs, respectively. For other immune markers (IDO, TIM-3, OX40, OX40L, B7-H2, B7-H3, B7-H4), immunohistochemical staining was identified in stromal TILs. In this study, we utilised two separate tissue microarray samples for positive and negative controls. Each antibody was applied to the 60 core-tissue microarray block, which contains 30 different human cancers from different organs (including skin, spleen, pancreas, liver, breast, etc.). After confirming positive and negative staining patterns of each immunohistochemical marker, the second 46 core-TMA block, composed of 23 invasive mammary carcinomas and paired normal breast tissues, were adopted to decide the best dilution point of each antibody in breast cancer tissue. Immunohistochemical staining was evaluated on the basis of stained location, stained percentage, and stained intensity of positively stained cells. Immunohistochemical staining were reviewed by two experienced breast pathologists (H.S.R. and M.S.J.) to ensure accuracy.

Immunohistochemical staining for ER and PR expression was categorised as positive when ≥1% of the tumour cells were stained according to the 2010 ASCO/CAP guidelines.^13^ Criteria of HER2-positive was assessed based on the 2013 ASCO/CAP guidelines.^14^ Patients were categorised as either ‘Luminal A’, ‘Luminal B’, ‘HER2-positive’, or ‘Triple negative’ according to the criteria of the 2011 St Gallen Consensus Panel.^15^ For 10 immune markers, immunohistochemical expression was measured by both intensity and proportion of the staining. The intensity of immunohistochemical staining (IS, intensity score) was graded as follows: 0 (negative), 1 (weak), 2 (moderate), 3 (strong). The proportion of immunohistochemical staining (PS, proportion score) was graded as follows: 0 (stain under <1%), 1 (1–5%), 2 (5–10%), 3 (10–25%), 4 (25 –50%), Grade 5 (>50%). Immune markers were defined as positive with one of the following; IS 1 with PS over 3, IS 2 with PS over 2, IS 3 with PS over 1. This cut-off is identical to Allred score (IS+PS) cut-off of 4 or higher (≥4) and H-score (IS*PS) cut-off of 3 or higher (≥3).

### Statistical analysis

The primary objective of this study was to investigate the effect of immune marker expression on patient survival (disease-free survival, DFS). Secondary endpoint was to elucidate the immunologic characteristics of breast cancer according to each subtype. The clinical database was last updated in July 2016. DFS was calculated from the date of operation to the first occurrence of one of the following events: recurrence of ipsilateral locoregional invasive breast tumour, contralateral invasive breast cancer, a distant disease recurrence, or death from any cause. Data from patients who were free of relapse or death were censored at the date of the last follow-up visit for DFS. Categorical variables were compared using chi-square test and continuous variables were compared using independent-samples T test. DFS were calculated using the Kaplan-Meier method and comparisons were made using the log-rank tests. Hazard ratios (HR) of immune markers were calculated using the Cox proportional hazard model. Baseline characteristics were adjusted by using a backward stepwise model including covariates that have a prognostic role: age (<60 vs.≥60), nuclear grade (1 and 2 vs. 3), histology grade (I and II vs. III), lymphovascular invasion, hormone receptor status (negative vs. positive), HER2 status (negative vs. positive), and tumour stage (I vs. II vs. III). Two-sided P-values of less than 0.05 were considered statistically significant. This study was a descriptive, explorative analysis and we did not perform multiple hypothesis comparison. Statistical analysis was performed with SPSS software for Windows, version 18.0 (SPSS, Chicago, IL, USA).

## Results

### Patient characteristics

A total of 392 breast cancer patients who received curative resection at SNUH were included. Baseline characteristics are summarised in Table 1. All patients were female with a median age of 47 (range: 27–77) years. Two hundred and one (69.1%) had luminal A disease, 36 (9.2%) had luminal B disease, 32 (8.2%) had HER2-positive disease, and 53 (13.5%) had triple negative disease. Tumour stage was I in 135 (34.4%) patients, II in 217 (55.4%) and III in 40 (10.2%). 305 (77.8%) patients received adjuvant chemotherapy, 282 (71.9%) received adjuvant hormone therapy and 259 (66.1%) received adjuvant radiotherapy. Among 56 patients with HER2(+), 46.9% (15/32) of HER2-positive disease and 33.3% (8/24) of HER2(+) luminal B patients received adjuvant HER2 directed therapy. According to the inclusion criteria, no patient received neo-adjuvant chemotherapy.

**Table 1:** Baseline characteristics according to immune recurrence score

Characteristics	Total (*N* *=* 392)	High-risk (*N* *=* 31)	Intermediate (*N* *=* 265)	Low-risk (*N* *=* 96)	*P*-value
*Age (years)*					
Mean (SD)	48.5 (9.8)	48.3 (9.2)	48.4 (10.2)	48.8 (8.9)	0.94
*Nuclear grade*					
1	8 (2.0%)	1 (3.2%)	4 (1.5%)	3 (3.1%)	0.028
2	159 (40.6%)	9 (29.0%)	99 (37.4%)	51 (53.1%)	
3	225 (57.4%)	21 (67.7%)	162 (61.1%)	42 (43.8%)	
*Histology grade*					
I	32 (8.2%)	1 (3.2%)	19 (7.2%)	12 (12.5%)	0.003
II	145 (37.0%)	8 (25.8%)	90 (34.0%)	47 (49.0%)	
III	215 (54.8%)	22 (71.0%)	156 (58.9%)	37 (38.5%)	
*Lymphovascular invasion*					
Absent	239 (61.0%)	14 (45.2%)	156 (58.9%)	69 (71.9%)	0.014
Present	153 (39.0%)	17 (54.8%)	109 (41.1%)	27 (28.1%)	
*Stage*					
I	135 (14.4%)	8 (25.8%)	80 (30.2%)	47 (49.0%)	0.009
II	217 (55.4%)	18 (58.1%)	159 (60.0%)	40 (41.7%)	
III	40 (10.2%)	5 (16.1%)	26 (9.8%)	9 (9.4%)	
*Hormone receptor*					
Negative	85 (21.7%)	12 (38.7%)	60 (22.6%)	13 (13.5%)	0.010
Positive	307 (78.3%)	19 (61.3%)	205 (77.4%)	83 (86.5%)	
*HER2*					
Negative	336 (85.7%)	24 (77.4%)	226 (85.3%)	86 (89.6%)	0.23
Positive	56 (14.3%)	7 (22.6%)	39 (14.7%)	10 (10.4%)	
*Intrinsic subtype*					
Luminal A	271 (69.1%)	14 (45.2%)	181 (68.3%)	76 (79.2%)	0.021
Luminal B	36 (9.2%)	5 (16.1%)	24 (9.1%)	7 (7.3%)	
HER2 positive	32 (8.2%)	3 (9.7%)	23 (8.7%)	6 (6.2%)	
Triple-negative	53 (13.5%)	9 (29.0%)	37 (14.0%)	7 (7.3%)	

*HER2* human epidermal growth factor receptor 2, *SD* standard deviation

### Immune marker and Immune recurrence score

Results of immunohistochemical staining of 10 immune markers are shown in Table 2. In tumour tissue, PD-L1 and PD-L2 was expressed in 3.8% and 60.5%, respectively. PD-1 was not expressed in tumour tissue. More than 30% of stromal TILs expressed B7-H3 (57.9%), B7-H2 (57.4%), OX40L (42.1%), OX40 (34.9%), and PD-1 (33.2%). Under 30% of stromal TILs expressed TIM-3 (28.3%), PD-L2 (27.8%), B7-H4 (27.0%), IDO (24.5%) and PD-L1 (12.0%).

**Table 2:** Immune marker expression according to intrinsic subtype

	Total	Luminal A	Luminal B	HER2-positive	Triple negative	*p–*value
	*N* (%)	*N* (%)	*N* (%)	*N* (%)	*N* (%)	
Total	392	271	36	32	53	
PD-1 (Tumour)	0 (0.0%)	0 (0.0%)	0 (0.0%)	0 (0.0%)	0 (0.0%)	–
PD-1 (TILs)	130 (33.2%)	66 (24.4%)	12 (33.3%)	20 (62.5%)	32 (60.4%)	<0.001
PD-L1 (Tumour)	15 (3.8%)	1 (0.4%)	1 (2.8%)	4 (12.5%)	9 (17.0%)	<0.001
PD-L1 (TILs)	47 (12.0%)	10 (3.7%)	5 (13.9%)	12 (37.5%)	20 (37.7%)	<0.001
PD-L2 (Tumour)	237 (60.5%)	161 (59.4%)	23 (63.9%)	23 (71.9%)	30 (56.6%)	0.50
PD-L2 (TILs)	109 (27.8%)	68 (25.1%)	10 (27.8%)	14 (43.8%)	17 (32.1%)	0.14
B7-H2	225 (57.4%)	159 (58.7%)	20 (55.6%)	18 (56.3%)	28 (52.8%)	0.87
B7-H3	227 (57.9%)	132 (48.7%)	28 (77.8%)	30 (93.8%)	37 (69.8%)	<0.001
B7-H4	106 (27.0%)	86 (31.7%)	8 (22.2%)	1 (3.1%)	11 (20.8%)	0.003
TIM3	111 (28.3%)	86 (31.7%)	13 (36.1%)	5 (15.6%)	7 (13.2%)	0.011
IDO	96 (24.5%)	66 (24.4%)	8 (22.2%)	5 (15.6%)	17 (32.1%)	0.38
OX40	137 (34.9%)	110 (40.6%)	12 (33.3%)	9 (28.1%)	6 (11.3%)	0.001
OX40L	165 (42.1%)	130 (48.0%)	16 (44.4%)	10 (31.3%)	9 (17.0%)	<0.001

Expression of immune markers were different among breast cancer intrinsic subtypes (Table 2). PD-1 (stromal TILs) and PD-L1 (Tumour and stromal TILS) were more expressed in HER2-positive and triple negative disease compared to luminal A and luminal B disease. In contrast, TIM-3, OX40 and OX40L were more commonly expressed in luminal A and luminal B disease. B7-H3 was most commonly expressed in HER2-positive disease. However, B7-H4 was detected in only 3.1% of HER2-positive disease.

After a median follow-up duration of 89 months, 50 recurrent events have occurred. The estimated 5-year DFS of the entire cohort was 89.1%. Influence of immune marker expression on DFS was evaluated. Prognostic role of each immune marker is shown in Table 3. Expression of OX40 (5-year DFS 92.4% vs. 87.4%, *p* = 0.036) and B7-H4 (5-year DFS 93.9% vs. 87.4%, *p* = 0.012) was associated with favourable DFS. In contrast, expression of B7-H3 (5-year DFS 86.7% vs. 92.4%, *p* = 0.027) was associated with worse DFS. Expression of PD-L1 (TILs) (5-year DFS 100.0% vs. 87.7%, *p* = 0.090), PD-L2 (TILs) (5-year DFS 93.2% vs. 87.6%, *p* = 0.055), OX40L (5-year DFS 91.2% vs. 87.6%, *p* = 0.053), and B7-H2 (5-year DFS 91.2% vs. 86.4%, *p* = 0.080) had a tendency of favourable DFS.

**Table 3:** Prognostic role of each immune marker

Immunologic marker		5-year DFS (%)	*p*-value
PD-1 (TILs)	Negative (*N* = 262)	88.2%	0.19
	Positive (*N* = 130)	91.0%	
PD-L1 (Tumour)	Negative (*N* = 377)	89.0%	0.55
	Positive (*N* = 15)	92.3%	
PD-L1 (TILs)	Negative (*N* = 345)	87.7%	0.090
	Positive (*N* = 47)	100.0%	
PD-L2 (Tumour)	Negative (*N* = 155)	90.5%	0.63
	Positive (*N* = 237)	88.2%	
PD-L2 (TILs)	Negative (*N* = 283)	87.6%	0.055
	Positive (*N* = 109)	93.2%	
B7-H2	Negative (*N* = 167)	86.4%	0.080
	Positive (*N* = 225)	91.2%	
B7-H3	Negative (*N* = 165)	92.4%	0.027
	Positive (*N* = 227)	86.7%	
B7-H4	Negative (*N* = 286)	87.4%	0.012
	Positive (*N* = 106)	93.9%	
TIM3	Negative (*N* = 281)	90.0%	0.48
	Positive (*N* = 111)	86.8%	
IDO	Negative (*N* = 296)	90.6%	0.27
	Positive (*N* = 96)	84.4%	
OX40	Negative (*N* = 255)	87.4%	0.036
	Positive (*N* = 137)	92.4%	
OX40L	Negative (*N* = 227)	87.6%	0.053
	Positive (*N* = 165)	91.2%	

To comprehensively analyse the prognostic role of multiple immune markers, we devised an immune recurrence score (IRS) using 7 immune markers (B7-H2, B7-H3, B7-H4, OX40, OX40L, PD-L1 and PD-L2) with prognostic value. Expression of each six immune markers with good prognosis (B7-H2, B7-H4, OX40, OX40L, PD-L1, and PD-L2) was counted as 1 and B7-H3 was counted as -1. The sum of 7 immune markers was calculated and was classified as follows: high-risk (IRS -1), intermediate-risk (IRS 0-2), or low-risk (IRS 3-6).

### Prognostic role according to immune recurrence score

Of the 392 patients, 31 (7.9%) were classified as IRS high-risk, 265 (67.6%) as intermediate-risk and 96 (24.5%) as low-risk. Baseline characteristics according to IRS is shown in Table 1. IRS high-risk group had higher proportion of patients with nuclear grade 3, histology grade III, lymphovascular invasion, stage III disease, hormone receptor positive disease and triple negative disease compared to intermediate and low-risk group.

**Fig. 1:**
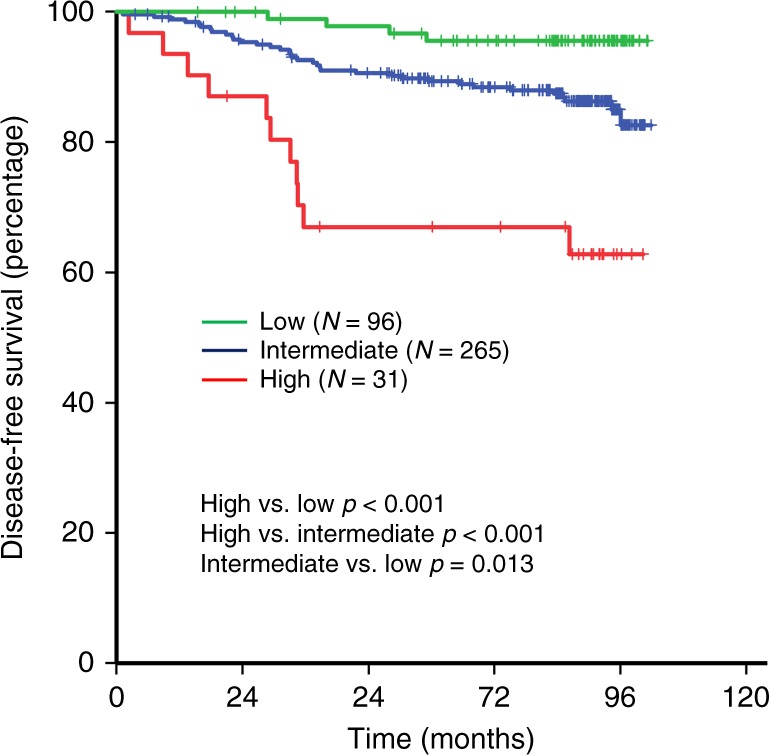
Correlation between immune recurrence score and disease-free survival

DFS was significantly worse in the high-risk group compared to intermediate and low-risk patients (5-year DFS 67.0, 89.4 and 95.6%, respectively) (Fig. 1). We performed multivariable analysis with a Cox proportional hazard model to examine whether IRS was independently associated with poor DFS. Multivariate analysis revealed IRS as an independent negative prognostic factor for DFS (Table 4). IRS low-risk group (adjusted HR for DFS, 0.14; 95% confidence interval [CI], 0.04–0.45; *p* *=* 0.001) and intermediate-risk group (adjusted HR for DFS, 0.32; 95% CI, 0.16–0.65; *p* *=* 0.002) had significantly lower risk of recurrence compared with high-risk group.

**Table 4:** Multivariate analysis of disease-free survival

		Adjusted HR (95% CI)	*p*-value
Age	≥ 60	2.31 (1.18–4.51)	0.015
	< 60	1	
Histology grade	III	4.38 (1.83–10.46)	0.001
	I or II	1	
Stage	III	5.03 (1.73–14.61)	0.012
	II	2.94 (1.14–7.59)	
	I	1.00	
Immune recurrence score	High	1	
	Intermediate	0.32 (0.16–0.65)	0.002
	Low	0.14 (0.04–0.45)	0.001

*HR* hazard ratio, *CI* confidence interval

**Fig. 2:**
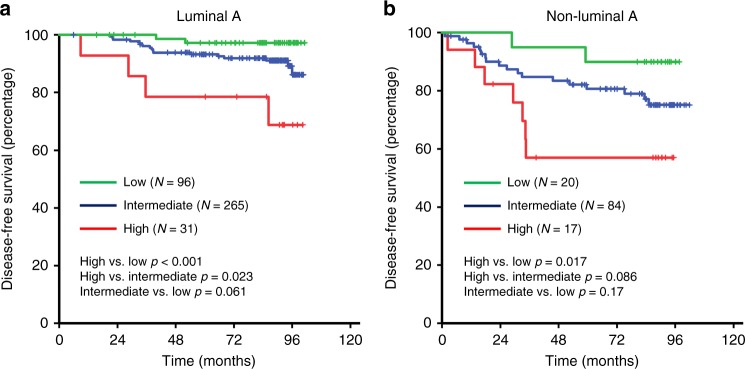
Prognostic role of immune recurrence score according to intrinsic subtype (**a** Luminal A, **b** Non-luminal A)

We next evaluated whether the prognostic role of IRS is persistent among each intrinsic subtype. Due to limited number of patients with luminal B, HER2-positive and triple negative disease, these patients were grouped into non-luminal A patients. IRS high-risk group was associated with worse DFS compared to intermediate-risk group and low-risk group in both luminal A patients (5-year DFS 78.6, 93.2 and 97.2%, respectively) and non-luminal A patients (5-year DFS 57.0, 80.7 and 90.0%, respectively) (Fig. 2). Multivariate analysis revealed IRS as an independent prognostic factor in both luminal A and non-luminal A patients (Supplement Table 1).

## Discussion

Cancer cells can evade immune destruction by obtaining immune tolerance through multiple mechanisms, including alteration of the immune checkpoint pathways.^16^ Immune checkpoint inhibitors targeting PD-1/PD-L1 can prolong survival in many types of cancer (including melanoma, non-small cell lung cancer, urothelial carcinoma, etc.) and is currently used in the clinic.^2–5^ Recently, atezolizumab plus nab-paclitaxel resulted in statistically significant PFS benefit for metastatic triple negative breast cancer patients as first line therapy.^6–8^ Although the PFS benefit in intention-to-treat patients were not translated into the overall survival benefit, the addition of atezolizumab showed clinically meaningful overall survival improvement in the PD-L1 positive population. It is speculated that the immunologic characteristics of tumours can affects its response to immune checkpoint inhibitors. Thus, there is an urgent need to elucidate immune characteristics of breast cancer and to discover predictive biomarkers of immunotherapy agents. This study identified immunologic characteristics of breast cancer patients using immunohistochemical staining of 10 immune markers. Moreover, we devised an IRS which can predict breast cancer recurrence.

In this study, we selected 10 immune markers with known/possible clinical relevance. PD-1 receptor is an immune check point, which plays a crucial role in tumour microenvironment.^17^ PD-L1 (B7-H1) is a main ligand for PD-1 and PD-L2 (B7-DC) is another ligand for PD-1.^17^ As previously noted, targeting PD-1 and PD-L1 has shown dramatic response in many types of tumour. B7 ligand family (B7-H2, B7H3, and B7H4) is an immune regulatory molecule which has a key role in regulating T lymphocyte activation at the peripheral tissue.^18^ B7-H2 is a co-stimulatory ligand for CD28, and B7-H4 function as a co-inhibitor of T-cell response.^18^ B7-H3 has a contradicting role that it may up-regulate but also down-regulate T-cell activity.^19^ TIM-3 is an immune checkpoint receptor which is expressed on CD4+ T cells and CD8+ T cells.^20^ Preclinical data show that TIM-3 may be a potential target for immunotherapy in cancer patients.^20^ IDO is a potent immune checkpoint, and recent evidences show that it may be a promising new target for immunotherapy.^21^ OX40 and its cognate ligand OX40L are expressed on activated T cells.^22^ They are upregulated in response to antigen presentation, and functions as a T-cell co-stimulatory molecule.^22^

In the present study, each breast cancer subtypes expressed distinct immunoregulatory protein. It is known that PD-L1 expression in tumour and TILs is higher in HER2-positive disease, ER negative disease and PR negative disease, respectively.^23^ However, it is not known whether the expression of other immune markers are different according to breast cancer subtype. In this study, luminal A and luminal B disease had higher expression of TIM-3, OX40, and OX40L in stromal TILs compared to HER2-positive and triple negative disease. In contrast, PD-1 (stromal TILs) and PD-L1 (both in tumour and stromal TILs) expression was higher in HER2-positive and triple negative disease. HER2-positive disease had high expression of B7-H3 (93.8%) but B7-H4 (3.1%) was rarely expressed. Expressions of other immune markers were similar between each tumour subtype.

Many types of immune cells infiltrate breast cancer microenvironment and each specific subsets of immune cells may suppress or activate antitumour responses.^1,9,24^ Despite lack of information on subpopulations, TILs have prognostic and predictive role in breast cancer patients, especially in triple negative disease.^9–11^ Although the prognostic value of TILs is concrete in triple negative disease, their role in luminal A or B disease is less significant. In the present study, expression of immunoregulatory protein by stromal TILs had prognostic role even in luminal A disease. Expression of B7-H3 by stromal TILs was associated with poor prognosis, and expression of B7-H4 and OX40 was associated with favourable prognosis. In addition, expression of PD-L1, PD-L2, B7-H2, and OX40L by stromal TILs had a tendency of better prognosis. Studies in many types of cancer show that B7-H3 might have a negative prognostic role.^25–27^ Expression of B7-H3 in prostate cancer was associated with poor survival and expression of B7-H3 in stage I to III breast cancer was associated with lymph node metastasis, which is a poor prognostic factor.^25,26^ While these studies did not evaluate B7-H3 expression by stromal TILs, expression of B7-H3 by stromal TILs may have negative prognostic role by modulating T-cell activity. B7-H4 is ubiquitously expressed in breast cancer (over 95%) and previous studies show that it may have negative prognostic role.^28,29^ However, the prognostic role of B7-H4 expression by stromal TILs has never been studied. Although B7-H4 expression by breast cancer may have negative prognostic impact, our study results show that expression of B7-H4 by stromal TILs may have a favourable prognosis. The prognostic role of B7-H2 is not known in breast cancer and our result show that it may have positive prognostic role in curatively resected breast cancer patients.^30^ As previously noted, OX40 and OX40L is expressed on activated T cells.^22^ Expression of OX40 and OX40L may have a positive prognostic role as these may reflect presence of an activated T cells. There are conflicting results on the prognostic role of PD-L1. In HER2-positive breast cancer patients, PD-L1 expression by tumour cell was associated with favourable prognosis while PD-L1 expression on TILs was not.^31^ In contrast, PD-L1 expression by TILs but not by tumour cell was associated with favourable prognosis in head and neck cancer patients.^32^ In the present study, PD-L1 and PD-L2 expression by TILs had a tendency of positive prognostic role while expression by tumour cells did not.

As immune cells act in a complex cross-talk, comprehensive analysis is important in identifying the prognostic role of immunoregulatory proteins. In the present study, we devised an IRS using 7 immune markers (B7-H2, B7-H3, B7-H4, OX40, OX40L, PD-L1 and PD-L2) with prognostic value. Multivariate analysis revealed IRS as an independent prognostic factor for DFS. IRS high-risk group had poor 5-year DFS compared to IRS intermediate-risk group and IRS low-risk group. These findings were consistent in both luminal A and non-luminal A patients. IRS might provide more comprehensive immunologic characteristics of breast cancer patients and may reflect microenvironment immunologic status. As stromal TILs have close interaction with tumours and regulate the tumour microenvironment immune system, immunoregulatory protein expression of TILs may be a potential predictive biomarker for immunotherapy. Current strategy to discover potent immunotherapy responder is limited to triple negative disease. However, our findings suggest that patients with different tumour subtype may have similar immunologic characteristics. We believe future work on the relationship between immunoregulatory protein expression of stromal TILs (including the IRS) and immunotherapy response is needed.

The limitation of the present study is that we could not quantify the amount of total stromal TILs as we used tissue microarray method. It is known that TILs have positive prognostic role in triple negative breast cancer patients. However, TILs are composed of heterogeneous mixture of various immune cell types. We believe immunohistochemical staining of 10 immune markers in stromal TILs can identify immunologic characteristics. Moreover, unlike TILs which do not have prognostic role in luminal A disease, IRS were able to predict recurrence in both luminal A and non-luminal A patients. Another limitation of the present study is that we did not have a validation cohort to confirm our findings. Although historical data support some of our findings, future work to validate our result is mandatory.

In conclusion, this study identified immunoregulatory protein expressions of breast cancer patients using 10 immune markers. In addition, we devised an IRS which could predict recurrence in stage I–III breast cancer. We believe our data have implications in future studies to develop predictive biomarkers for immunotherapy in breast cancer and help physicians to predict breast cancer recurrence.

## Supplementary information

Supplement Table 1

## Data Availability

The datasets used and/or analysed during the current study are available from the corresponding author on reasonable request.
