# Supplementary material for: Immune recurrence score using 7 immunoregulatory protein expressions can predict recurrence in stage I–III breast cancer patients
Source: Br J Cancer. 2019 Jul 11;121(3):230–6. doi: 10.1038/s41416-019-0511-9 (PMC6738097; doi:10.1038/s41416-019-0511-9)
Supplement: Supplementary file 1 — Supplement Table 1 [file 41416_2019_511_MOESM1_ESM.docx]

**Supplement Table 1. Multivariate analysis according to intrinsic subtype**

| **Supplement Table 1. Multivariate analysis according to intrinsic subtype** | | | |
| --- | --- | --- | --- |
| **Luminal A** |  | **Adjusted HR (95% CI)** | ***p*-value** |
| Age | ≥ 60 | 4.43 (1.76 – 11.15) | 0.002 |
|  | < 60 | 1 |  |
| Histology grade | III | 2.93 (1.11 – 7.74) | 0.030 |
|  | I or II | 1 |  |
| Stage | III | 6.46 (1.20 – 34.79) | 0.030 |
|  | II | 3.61 (0.80-16.29) | 0.095 |
|  | I | 1 |  |
| Immune recurrence score | High | 1 |  |
|  | Intermediate | 0.26 (0.08 – 0.85) | 0.026 |
|  | Low | 0.15 (0.03 – 0.86) | 0.033 |
|  | | | |
| **Non-Luminal A** |  | **Adjusted HR (95% CI)** | ***p*-value** |
| Hormone receptor | Positive | 1.98 (0.90 – 4.36) | 0.090 |
|  | Negative | 1 |  |
| Lymphovascular invasion | Present | 2.42 (1.10 – 5.31) | 0.028 |
|  | Absent | 1 |  |
| Immune recurrence score | High | 1 |  |
|  | Intermediate | 0.39 (0.16 – 0.95) | 0.038 |
|  | Low | 0.16 (0.03 – 0.77) | 0.022 |
